# Patterns of primary care among persons with schizophrenia: the role of patients, general practitioners and centre factors

**DOI:** 10.1186/s13033-020-00409-z

**Published:** 2020-11-10

**Authors:** Mª Carmen Castillejos Anguiano, Carlos Martín Pérez, Antonio Bordallo Aragón, Jesus Sepúlveda Muñoz, Berta Moreno Küstner

**Affiliations:** 1 Departament of Personality, Assessment and Psychological Treatment, Faculty of Psychology, Andalusian Group of Psychosocial Research (GAP), Biomedical Research Institute of Malaga (IBIMA), University of Malaga, Campus Teatinos, 29071 Malaga, Spain; 2 Clinical Management Unit At Marquesado, Andalusian Health Service, Carretera Los Pozos, North East Granada Sanitary District, 18518 AlquifeGranada, Spain; 3 Clinical Management Unit of Mental Health of the Regional Hospital of Malaga, Andalusian Health Service, Paseo Limonar, Malaga, Spain; 4 Alameda-Perchel Basic Primary Care Team, Health District Malaga-Guadalhorce, Andalusian Health Service, Avenida Manuel Agustín Heredia, Malaga, Spain

**Keywords:** Primary health care, Schizophrenia, Use of services

## Abstract

**Background:**

Patients with schizophrenia and related disorders have more physical problems than the general population. Primary care professionals play an important role in the care of these patients as they are the main entry point into the healthcare system. We aimed to identify patient, general practitioner, and primary care centre factors associated with the number of visits of patients with schizophrenia and related disorders to general practitioners.

**Methods:**

A descriptive, cross-sectional study was conducted in 13 primary care centres belonging to the Clinical Management Unit of Mental Health of the Regional Hospital of Málaga, Spain. The eligible population was composed of patients with schizophrenia and related disorders attending the primary care centres in the study area, and the general practitioners who attend these patients. Our dependent variable was the total number of general practitioner visits made by patients with schizophrenia and related disorders during the 3.5-year observation period. The independent variables were grouped into three: (a) patient variables (sociodemographic and clinical), (b) general practitioner variables, and (c) primary care centre characteristics. Descriptive, bivariate, and multivariate analyses using the random forest method were performed.

**Results:**

A total of 259 patients with schizophrenia and related disorders, 96 general practitioners, and 13 primary care centres were included. The annual mean was 3.9 visits per patient. The results showed that younger general practitioners, patients who were women, patients who were married, some primary care centres to which the patient belonged, taking antipsychotic medication, presenting any cardiovascular risk factor, and more frequency of mental health training sessions at the primary care centre were associated with an increased number of visits to general practitioners.

**Conclusions:**

The only general practitioner variable that was associated with the number of visits was age, the older the less contact. There were also patient variables involved in the number of visits. Finally, mental health training for general practitioners was important for these professionals to manage patients with schizophrenia and related disorders.

## Background

Patients with schizophrenia have high levels of medical comorbidity and cardiovascular risk factors [1, 2]. Thus, the lifespan of these patients with severe mental illness (SMI) is shorter compared to the general population [3]. This is due not only to their particular lifestyle [4–8] but also to the antipsychotic medication they are taking [8–10].

Primary care has multiple benefits for patients in general and for patients with a complex chronic illness specifically, and a relationship has been demonstrated between availability of primary care and survival in patients with schizophrenia and related disorders (SRDs) [11]. However, the attention of these patients to their physical problems by primary care continues to be deficient. In previous work, it was found that patients with SRDs had significantly lower recorded rates of cardiovascular disorders [12, 13].

It seems that general practitioners (GPs) stigmatise mental illnesses more than psychiatrists when the attitudes of different categories of health professionals were compared [14–16]. This perception could influence their decisions in daily practice [17], which can be detected by patients [18]. In addition, feeling unprepared, having low confidence, and a lack of resources to manage mental illness from GPs [19, 20] could encourage GPs to transfer these patients to specialised care [21]. Both factors could explain the low contact of patients with SRDs with primary care services [16, 21, 22]. On the other hand, mental health professionals often do not feel confident in prescribing physical health medication [23]. However, it has been seen that when there was a collaborative work model between mental health and primary care services, GPs felt more confidence in managing patients with SMI [21], had fewer misperceptions of mental illness, and were less inclined to stigmatise it [24].

Therefore, the excess of mortality of patient with SRDs is due not only to their lifestyle and antipsychotic medication but also to poorer access to and quality of received health care, negative perceptions of people with SMI, and lack of awareness of increased cardiovascular risk in these patients by some health professionals [3, 25]. Taking this into account and bearing in mind the important role of GPs as a main entry point into the healthcare system and as specialists in physical health problems, it is essential to study whether the characteristics of these professionals could influence the care of patients with SRDs. In a previous study, we analysed the association between patient factors and primary care centre (PCC) variables with the number of SRD patient visits to GPs [22], but we lacked an important link—the role of GP characteristics in the contact. Therefore, in the present study we aimed to identify patient, general practitioner, and primary care centre factors associated with the number of visits of patients with schizophrenia and related disorders to general practitioners.

## Methods

An observational, cross-sectional study was conducted.

### Study area and temporal scope

The study was carried out in the Community Mental Unit of Mental Health (CMU-MH) of the Regional Hospital of Málaga, with two Community Mental Health Units (CMHUs), Central and Northern, which together covered a population of 315,159 inhabitants. The Central CMHU included six PCCs: Alameda-Perchel, Victoria, Limonar, El Palo, Colmenar, and Rincón de la Victoria; and the Northern CMHU included seven PCCs: Trinidad, Nueva Málaga, Miraflores, Palma-Pamilla, Ciudad-Jardín, Capuchinos, and Carlinda.

The study information was collected from January 1, 2008 to July 1, 2011.

### Eligible population and sample

#### Patients

The eligible population was composed of the patients with SRDs included in the Málaga Schizophrenia Case Register (RESMA). The RESMA was a case register of patients with SRDs who were attending the CMU-MH Area Regional Hospital of Málaga to improve the care of patients with SMI [26].

The sample size was calculated to be representative of the patients of each PCC. Patients were selected with simple random sampling, stratified by PCC. Inclusion criteria were: (a) age over 14 years, (b) having a clinical diagnosis of SRD according to the ICD-10 before January 1, 2008, and (c) being in contact with a PCC in the coverage area of the CMU-MH of the Regional Hospital of Málaga. Exclusion criteria were: (a) being in treatment outside the study area, (b) having died during the follow-up, and (c) not having a computerized medical history in their reference PCC. In the present study only patients who had information about their GP were included (N = 259). This is because one of our objectives was to analyse the GPs characteristics.

#### General practitioners

We obtained information from 96 GPs in total, all of whom worked in the study area and attended patients with schizophrenia included in our study.

### Study variables

Our dependent variable was the total number of GP visits made by patients with SRD during the three-and-a-half-year observation period.

The independent variables were grouped into three groups:

- Patient variables. Sociodemographic: gender; marital status; educational level; living arrangements; employment status; type of area where they lived; whether they lived in a socioeconomically deprived area; primary care centre which they belonged to; and age. Clinical variables: ICD-10 clinical diagnosis; global level of severity; cardiovascular risk factors; and taking antipsychotic medication. (Additional file 1: Table S1).
- GPs variables. Gender; specialization training as general practitioner; accredited training as a tutor; having any residential training students; age; time to complete the medical degree; size of patient list; relationship; training; and beliefs. (Additional file 1: Table S1).
  We used the Primary Care Physicians and Mental Health Questionnaire (MAPSAM-14), which has been validated by the research team [27], to assess perceptions of GPs towards mental health. This instrument offers scores on the three MAPSAM-14 scales: (1) Relationship: the level of satisfaction of GP’s relationship with the local CMHU (range: 7–15; higher scores indicated greater satisfaction with the relationship); (2) Beliefs: that touched upon erroneous beliefs, stigmas and attitudes about mental illness and stigmatisation of mental illness (range: 5–12; higher scores indicated more erroneous beliefs and greater stigmatisation); (3) Training: the GP’s perception of the adequacy of his or her training in mental health, including schizophrenia and related disorders (range: 5–15; higher scores indicated greater perceived adequacy of training).
- Primary care centre variables: hometown; frequency of mental health care visits; frequency of mental health training session; training from mental health services; active role of GPs, nurses and social workers; and level of communication between primary care centre and community mental health centre, as well as level of communication between GPs and nurses; and communication between GPs and social workers (Additional file 1: Table S1).”

### Data sources

Data on the total number of GP visits were obtained from digitised primary care records (DIRAYA program). Sociodemographic and clinical information about patients was obtained from RESMA [26, 28]. Information about the PCCs was collected from their directors, who were interviewed using a questionnaire designed by the research team of this study. Finally, GPs filled in another questionnaire also designed by the research team [28].

### Data analysis

The categorical variables used in the descriptive analysis were frequency distribution and percentages. Descriptive statistics (mean, standard deviation, and median) were calculated for the continuous variables.

We used a bivariate analysis to examine the relationships between the dependent (number of GP visits) and independent (predictive) variables. For categorical variables with two categories, the Wilcoxon test was used, and for categorical variables with more than two categories, the Kruskal–Wallis test was used. Finally, for continuous explanatory variables, Spearman’s correlation coefficient was calculated. Differences were considered significant at p < 0.05.

The classic multivariate analysis (linear regression) is hindered by the large number of predictor variables in relation to the relatively small sample size of the study. We used the random forest technique to solve this problem.

Random forest (RF) is one of the so-called ensemble methods for predictive regression models, which is based on the methodology of classification and regression trees (CART). With the RF procedure, a large group of trees is generated in which each tree predicts the result of the dependent variable. In the process of constructing the set of trees, RF uses two types of randomization. First, each tree is constructed using a subsample of the database. The size is approximately two-thirds of the initial sample. In addition, a second level of randomness is added by selecting for each tree a random sample of the predictive variables in each node to choose the best division.

The number of predictors selected in each node and the total number of trees that are built are the two main parameters of the RF algorithm. A third parameter is the minimum size of each node terminal.

As a result of the use of a subsample of training data, about one-third of the samples are omitted when building each tree. These are the so-called out-of-bag samples (OOB), which can be used to evaluate the prediction error and to construct measures of importance of the predictive variables.

We used the permutation index of importance to evaluate the importance of the predictive variables. The importance of a variable was assessed by estimating the change in the prediction error that occurs when, in the OOB data, the variable is randomly swapped while the others remain unchanged. The calculations were made tree-by-tree as the random forest was constructed. If a variable was important in the problem analysed, changing its random values led to larger changes in the prediction adjustment compared to those variables that were not important.

The variables to be included in the model were selected so that it explained the highest percentage of the variability with respect to the result, assuming the least possible prediction error. To do this selection, the VSURF package in the R software was used, which followed a two-step strategy. First, the variables of the initial model (all) were classified according to their importance, and those that did not exceed a threshold were eliminated, which was determined based on the standard deviation of that importance. In a second step, with the variables selected in the previous step, a series of nested RF models were built, and the one that generated a lower OOB error was selected. The variables selected will be important and will have a strong relationship with the dependent variable.

For missing data that did not exceed 15% of the total data of the variable, the miss forest package in R was used, which can be used to impute continuous and/or categorical data including complex interactions and nonlinear relationships. Variables presenting more than 15% of missing data were eliminated.

We used the Random Forest R package and its default parameters, and then these hyper parameters were tuned to obtain the least possible error.

The statistical package R was used for the statistical analysis.

## Results

Of the total sample of 494 patients included in the study carried out by Castillejos et al. [22], 259 were included. Therefore, the total sample in the present study consisted of 259 patients and 96 GPs. Regarding the patients included, the sociodemographic profile was as follows. The mean age at the beginning of the study was 43.88 years (range: 22‒80); 63.71% were male; 70.66% were single; 43.63% completed only primary school; 55.21% were living with their original family or other relatives; 37.84% were unable to work; 88.03% were living in urban areas; and 89.19% were living in non-socioeconomically-deprived areas. Most patients had a clinical diagnosis of schizophrenia (69.5%), 82.24% of whom had a low or medium global level of severity, 39.23% had at least one cardiovascular risk factor (type 2 diabetes mellitus, hypertension, hypercholesterolaemia, obesity and smoking), and 69.11% were taking antipsychotic medication (see Table 1).

**Table 1:** Characteristics of patients with schizophrenia and related disorders

Sociodemographic characteristics	Patients N (%)
Gender	
Male	165 (63.71%)
Female	94 (36.29%)
Marital status	
Single	183 (70.66%)
Married/civil partnership/cohabiting	47 (18.15%)
Separated/divorced/widowed	29 (11.2%)
Educational level	
No formal education and/or illiterate	45 (17.37%)
Primary school	113 (43.63%)
Secondary school	72 (27.8%)
Higher education (Bachelor’s degree)	29 (11.2%)
Living arrangements	
Alone	33 (12.74%)
Original family/other relatives or friends	143 (55.21%)
Own family	57 (22.01%)
Sheltered accommodation	23 (8.88%)
Homeless	3 (1.16%)
Employment status	
Employed	46 (17.76%)
Unemployed	49 (18.92%)
Student	16 (6.18%)
Carer or househusband/housewife	14 (5.41%)
Not working, receiving welfare benefits	98 (37.84%)
Other	36 (13.9%)
Type of area	
Urban	228 (88.03%)
Rural	31 (11.97%)
Within a socioeconomically deprived area	
No	231 (89.19%)
Yes	28 (10.81%)
Primary care centre	
Trinidad	21 (8.11%)
Nueva Málaga	7 (2.7%)
Miraflores	10 (3.86%)
Palma-Palmilla	17 (6.56%)
Ciudad Jardín	25 (9.65%)
Capuchinos	8 (3.09%)
Carlinda	3 (1.16%)
Alameda Perchel	26 (10.04%)
Victoria	33 (12.74%)
Limonar	30 (11.58%)
El Palo	50 (19.31%)
Rincón de la Victoria	28 (10.81%)
Colmenar	1 (0.39%)
Clinical characteristics	
ICD-10 Clinical diagnosis	
F20 Schizophrenia	180 (69.5%)
F22 Persistent delusional disorders	26 (10.04%)
F23 Acute and transient psychotic disorders	25 (9.65%)
F25 Schizoaffective disorders	17 (6.56%)
F21, F24, F28, F29 Schizotypal disorder, Induced delusional disorder, other non-organic psychotic disorders and unspecified non-organic psychosis	11 (4.25%)
Global level of severity	
Level I (low severity)	91 (35.14%)
Level II	122 (47.1%)
Level III (high severity)	46 (17.76%)
Cardiovascular risk factors	
No	156 (60.23%)
Yes	103 (39.77%)
Taking antipsychotic medication	
No	80 (30.89%)
Yes	179 (69.11%)
Total	259 (100%)

Categorical variables (N = 259)

Regarding characteristics of GPs, the mean age at the beginning of the study was 50.84 years old (range: 38‒63), 63.54% were women, about 50% had specialised training as a GP, 65.62% were not accredited training as a tutor, 63.16% had a residential training student, and 87.5% had training from mental health services. Moreover, it took 4‒11 years to finish their medical degree. The size of the patient list varied from 900 to 2,434 patients per GP. Regarding the perception of GPs on mental illness, they presented high punctuation in the level of satisfaction with their relationship with the community mental health centre, medium punctuation in the perception of their level of training in mental health, schizophrenia, and other psychotic disorders, and very low punctuation in erroneous beliefs and stigmatisation about mental illness (see Table 2).

**Table 2:** Characteristics of general practitioners (N = 96)

Variable	N (%)	Minimum	Maximum	Mean	Median
Gender					
Male	35 (36.46%)				
Female	61 (63.54%)				
Specialisation training as a General Practitioner (GP)					
Yes	53 (56.38%)				
No	41 (43.62%)				
Accredited training as a tutor					
Yes	33 (34.38%)				
No	63 (65.62%)				
Having any residential training student					
Yes	60 (63.16%)				
No	35 (36.84%)				
Age		38	63	50.84	52
Time to complete the medical degree		4	11	7.49	7
Size of patient list		900	2434	1603	1500
Relationship^a^		7	15	12.71	13
Training^b^		4	11	8.1	8
Beliefs^c^		4	10	5.05	4

^a^Level of satisfaction of GPs with their relationship with the community mental health centre

^b^The GPs’ perception of their level of training in mental health, schizophrenia and other psychotic disorders

^c^Erroneous beliefs, stigmas and attitudes regarding mental illness

With respect to PCC variables, 69.2% of the directors of the PCCs agreed that PCCs received assistance visits from mental health services more than twice a month, and 46.2% of them agreed that PCCs received training sessions from mental health services once a month or more. Most of the directors (76.9%) agreed that there was training from mental health services to PCCs. In addition, most of the directors agreed that social workers (69.3%) play an active role in managing patients’ mental health, but nevertheless, 53.9 and 36.46% of them agreed with this role in GPs and nurses, respectively. Regarding communication between the PCC and community mental health centre, almost all directors interviewed (92.71%) agreed with it was good or very good. All of them agreed with the good communication between GPs and nurses, and 69.3% agreed with the good communication between GPs and social workers (see Table 3).

**Table 3:** Primary care centre variables N = 13

	N (%)
Hometown	
2,500–5,000 inhabitants	1 (7.7%)
5,000–10,000 inhabitants	1 (7.7%)
10,000–15,000 inhabitants	1 (7.7%)
15,000–20,000 inhabitants	1 (7.7%)
20,000–30,000 inhabitants	6 (46%)
More than 30,000 inhabitants	3 (23.1%)
Frequency of mental health care visits in primary care centres	
Twice a month	4 (30.8%)
More than twice a month	9(69.2%)
Frequency of mental health training sessions in primary care centres	
None	3 (13.23.1%)
Once a year or less	1 (7.7%)
Between 4 and 6 months	1 (7.7%)
Every 2 months	2 (15.4%)
Once a month	4 (30.8%)
Twice a month	1 (7.7%)
More than twice a month	1 (7.7%)
Training from mental health services	
Yes	10 (76.9%)
No	3 (23.1%)
Primary care physician active role in managing patients’ mental health	
Neither agree nor disagree	6 (46.2%)
Agree	5 (38.5%)
Completely agree	2 (15.4%)
Nurse active role in managing patients’ mental health	
Completely disagree	1 (7.7%)
Neither agree nor disagree	7 (53.8%)
Agree	2 (15.4%)
Completely agree	3 (23.1%)
Social worker active role in managing patients’ mental health	
Disagree	1 (7.7%)
Neither agree nor disagree	2 (15.4%)
Agree	4 (30.8%)
Completely agree	5 (38.5%)
Level of communication between the primary care centre and community mental health centre	
Neither good nor bad	1 (7.7%)
Good	6 (46.2%)
Very goodW	6 (46.2%)
Level of communication between primary care physicians and nurses	
Good	11 (84.6%)
Very good	2 (15.4%)
Level of communication between primary care physicians and social workers	
Neither good nor bad	3 (23.1%)
Good	6 (46.2%)
Very good	3 (23.1%)

The mean number of patient contacts with their GPs during the study period was 13.53 (median = 11; range: 0‒69). The annual mean was 3.9 visits per patient.

The bivariate analysis showed that the patient factors associated with more contact with GPs were: being a woman (p ≤ 0.001), being married or with a partner (p = 0.0038), having no formal education (p = 0.011), having a schizoaffective disorder diagnosis (p = 0.0213), having any cardiovascular risk factor (p ≤ 0.001), and taking antipsychotic medication (p = 0.0011; see Tables 4 and 5).

**Table 4:** Bivariate analysis between dichotomous variables and number of contact with general practitioners

	W Wilconxon	P value
Patient gender	10,172	≤ *0.001*
Cardiovascular risk factors	5380	≤ *0.001*
Taking antipsychotic medication	5352	*0.0011*
Type of area	4005.5	0.2284
Within a socioeconomically deprived are	3005	0.5413
General Practitioner gender	8797.5	0.0720
Specialisation training as a General Practitioner	9216.5	0.0718
Accredited training as a tutor	8662.5	0.0653
Having any residential training student	8697	0.1118
Training from mental health services	3166	0.1055
Level of communication between primary care physicians and nurses	2343	0.8850

**Table 5:** Bivariate analysis between categorical variables with more than two categories and number of contact with general practitioners

	Chi squared	df	P value
Patient marital status	11.11	2	*0.0038*
Patient educational level	11.246	3	*0.0110*
Patient living arrangements	4.650	4	0.3251
Patient employment status	5.446	5	0.3639
Primary care centre	24.036	12	*0.0201*
ICD-10 Clinical diagnosis	11.518	4	*0.0213*
Global level of severity	0.689	2	0.7086
Hometown	4.6082	5	0.4655
Primary care physician active role in managing patients’ mental health	5.874	2	0.0530
Nurse active role in managing patients’ mental health	7.446	3	0.0589
Social worker active role in managing patients’ mental health	6.760	4	0.1491
Level of communication between the primary care centre and community mental health centre	4.490	2	0.1059
Level of communication between primary care physicians and social workers	7.778	2	0.0508

In addition, the GP factors that were clearly associated with more contact with GPs were as follows: GP age was negatively associated, as older GPs were associated with less contact (p ≤ 0.001); and the training variables were also positively associated, such that GPs who had a perception of higher level of training in mental health received more contact (p = 0.0219; see Table 6).

**Table 6:** Bivariate analysis between continuous variables and number of contact with general practitioners

	ρ	p value
Patient age	0.098	0.1162
General practitioner age	−0.247	≤ *0.001*
Time to complete the Medical degree	0.058	0.3504
Size of patient list	−0.060	0.3610
Relationship	0.053	0.3945
Training	0.124	*0.0219*
Beliefs	−0.111	0.0740

Lastly, there were statistically significant differences among the PCCs to which patients attended (p = 0.02). Colmenar (with a mean contact of 33), Trinidad (17.8), El Palo (17.5), and Carlinda (15) were the PCCs that presented more visits to the GPs (see Table 6). There were others PCC factors that almost reached statistical significance: GP and nurse roles in managing patients’ mental health, such that higher punctuation in GP role was positively associated with amount of contact (p = 0.053), but higher punctuation in nurse role was negatively associated (p = 0.0589); and communication between GPs and social workers was positively associated with amount of contact with GPs (p = 0.0508; see Additional file 2: Table S2).

**Fig. 1:**
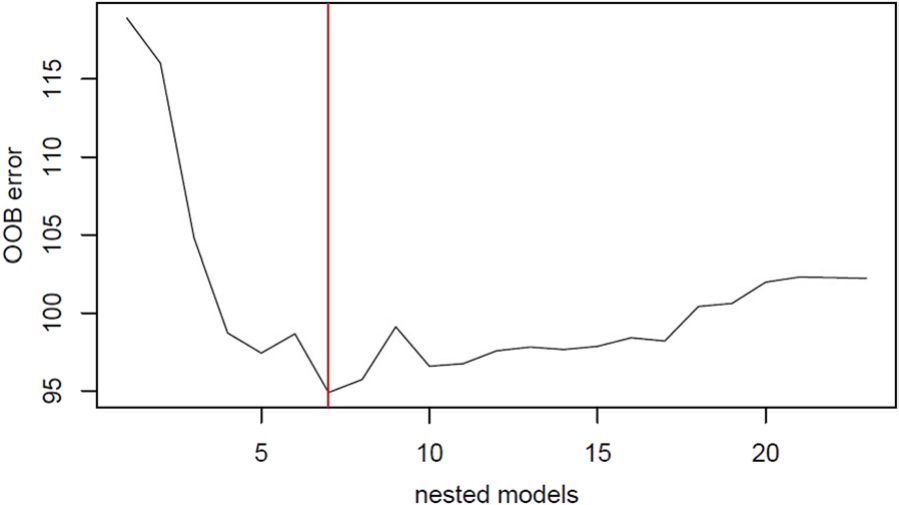
OOB error variation depending on the different nested models

**Fig. 2:**
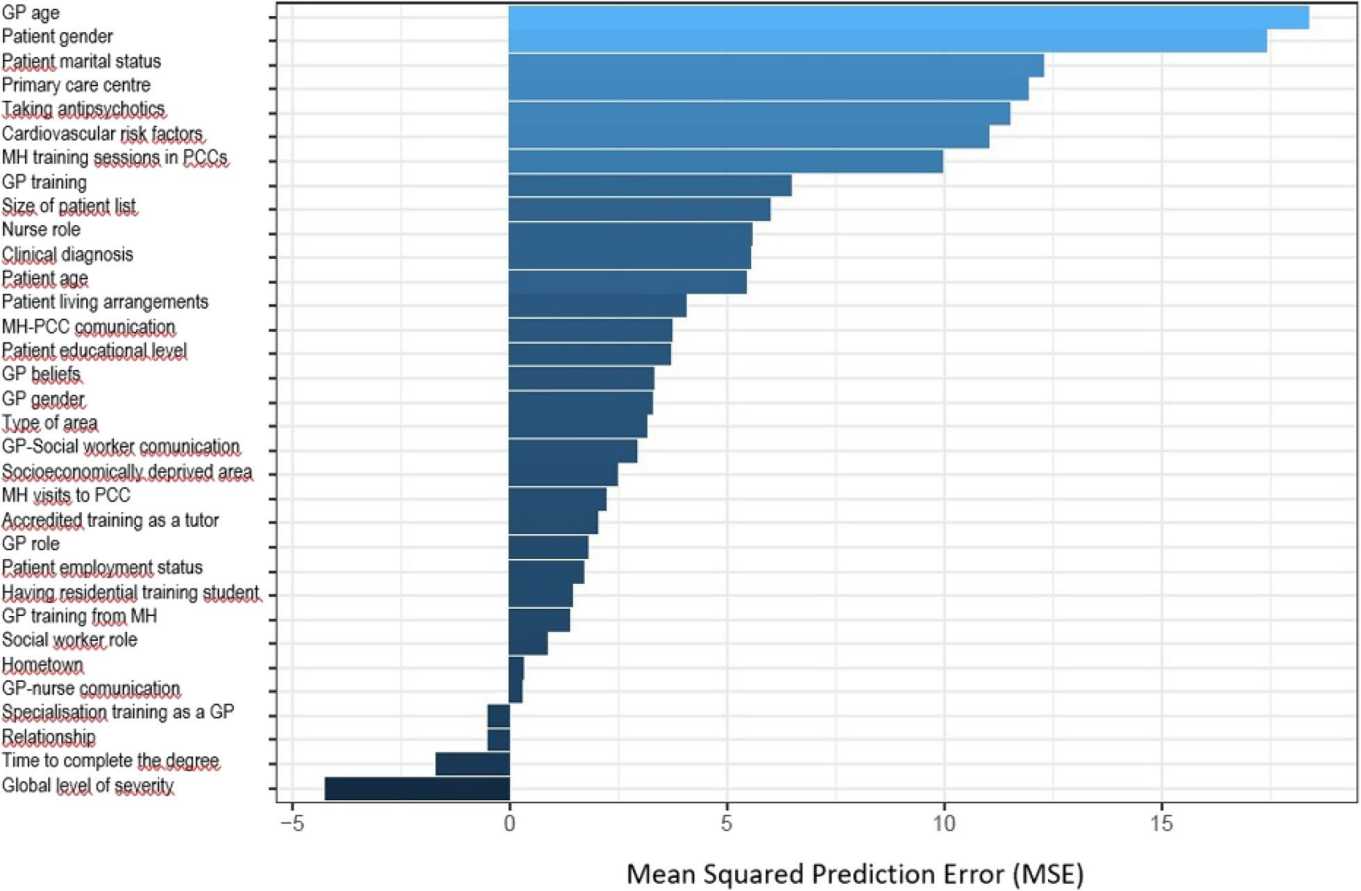
Mean Square Prediction Error depending on the importance by permutation of the variables of the initial model. *GP* general practitioner, *MH* mental health, *PCC* Primary Care Centre

Regarding the multivariate analysis, based on the method explained previously in the data analysis, we achieved the model with the lowest OOB error with seven variables (see Fig. 1). Therefore, younger general practitioners, patients who were women, patients who were married, some primary care centres to which the patient belonged, taking antipsychotic medication, presenting any cardiovascular risk factor, and more frequency of mental health training sessions at the primary care centre were strongly associated with an increase in the number of visits to GPs in the multivariate analysis (see Fig. 2).

## Discussion

In a previous study carried out by our research group, we analysed the relationship between patients with SRD/PCC characteristics and the number of visits to GPs [22]. The novelty of the present study is in the association of characteristics of the GPs who treated these patients with the amount of contact with GPs, which led to important findings. GP age, patient gender, patient marital status, PCC to which the patient belonged, taking antipsychotic medication, presenting any cardiovascular risk factor, and frequency of mental health training visits to the PCC were associated with an increased number of visits to GPs. In addition, a novel statistical analysis technique based on the random forest method was used to perform a multivariate analysis.

With the subsample analysed, the mean annual number of visits to GPs during the observation period was similar to that found with the entire sample analysed and discussed in the previous study [22].

The variable that was most associated with the number of visits was GP age, such that older GPs had less contact with patients. This could be related to older GPs having a more negative attitude toward patients with schizophrenia than their younger counterparts [29], although not all studies have found this result [24, 30].

Women visited GPs more often, which is in line with other studies [11, 31, 32].

Patient marital status was also related to the number of visits to GPs, so that patients who were married or living with a partner visited GPs more than single patients. This could be because family support causes these patients to receive continued care. We also found that patients who were separated, divorced, or widowed made more visits to their GPs than single patients. Copeland et al. [11] found that the number of visits to GPs by married patients or those with a partner was greater, but no greater number of these visits was found in patients who were separated, divorced, or widowed.

The fourth variable that was more related to contact with GPs was the PCC to which patients belonged. In a previous study performed by our group, we found that GPs working at PCCs in the catchment area of Central CMHU had less stigma about mental illness, coinciding with the fact that this area had been working collaboratively with mental health services for 15 years [24]. However, in this study we found that the PCCs that received the highest number of visits to GPs belonged to both Central and Northern CMHU. These two facts seem to indicate that the stigma presented by GPs toward mental illness was not related to the number of visits they received from patients with SRDs. In addition, working in a collaborative model was not related to the increase in contact with GPs, which is in line with the results found in a previous study in which good communication between primary care and mental health services did not translate into increased contact, and this might be due to mental health professionals assuming some of the functions that otherwise would have been performed by the GPs [24].

Moreover, taking antipsychotic medication and presenting cardiovascular risk factors were also associated with a higher number of visits to GPs. On one hand, the fact that GPs are the health professionals in charge of the follow-up with diseases such as cardiovascular risk factors mean that patients with SRDs who present these problems visit their GP more than those who do not present them. On the other hand, GPs are also in charge of the follow-up in prescribing medication, hence both patients taking antipsychotic medication and those with cardiovascular risk factors (who usually also take medication for this disease) visit their GPs more frequently.

Finally, higher frequency of mental health training visits to the PCC resulted in more visits to the GP. Related to this is that, in the bivariate analysis, GP perception of higher level of training in mental health received more contact. These results are in line with the study carried out by Bagayogo et al. [20], who found that GP felt low confidence in treating complex mental health problems and the need to integrate mental health into primary care. Moreover, it was also found that when there was a collaborative work model between mental health and primary care services, GPs felt more confidence in managing patients with severe mental illness [21]. Therefore, it seems that a collaborative work model between mental health and primary care services at the training level can make GPs feel more capable of managing these patient problems and refer less to specialised services.

### Strengths and limitations

- This study analysed multiple associations between patient, PCC, and GP characteristics and the number of visits by SRD patients to GPs.
- The study was carried out in a wide catchment area, including the participation of 13 PCCs.
- We analysed a large, homogeneous sample of patients diagnosed with SRDs living in the community.
- We performed a multivariate analysis using a novel statistical analysis technique based on the random forest method.
- For unknown reasons, not all GPs in the study area participated, and in some cases, the number of GPs per centre was very low. However, in general, the participation rate was high.
- This was a cross-sectional study from which we cannot infer causality but only association.
- Clinical diagnoses were not made through clinical interviews but rather by the patients’ long-term psychiatrists and updated in the RESMA database.
- Information about patients was collected from medical records instead of specific health screening tests of individual patients.

## Conclusions

This study analysed multiple associations between patient, GP, and PCC characteristics and the number of visits by SRD patients to GPs. It was surprising that the only GP characteristic that was clearly associated with the number of visits was GP age. It was confirmed that there were patient variables involved in the number of visits, such as gender and marital status. Finally, it was shown that the training of GPs is important for these professionals to manage patients with SRDs. Therefore, a collaborative model between mental health and primary care services should be promoted at the training level.

## Supplementary information

**Additional file 1: Table S1.** Study variables.

**Additional file 2: Table S2.** Categorical variables associated with the number of visits to general practitioners.

## Data Availability

The data that support the findings of this study are available from Berta Moreno Küstner but restrictions apply to the availability of these data, which were used under license for the current study, and so are not publicly available. Data are however available from the authors upon reasonable request and with permission of Berta Moreno Küstner.

## Abbreviations

CART: Classification and regression trees
CI: Confidence Interval
CMU-MH: Clinical Management Unit of Mental Health
CVRF: Cardiovascular Risk Factors
GLS: Global Level of Severity
GP: General Practitioner
ICD: International Classification of Diseases
OOB: Out of bag
PCC: Primary Care Centre
RESMA: Malaga Schizophrenia Case Register
RF: Random forest
SMI: Severe mental illness
SRD: Schizophrenia and related disorders
