# Supplementary material for: Patterns of primary care among persons with schizophrenia: the role of patients, general practitioners and centre factors
Source: Int J Ment Health Syst. 2020 Nov 10;14:82. doi: 10.1186/s13033-020-00409-z (PMC7653995; doi:10.1186/s13033-020-00409-z)
Supplement: Supplementary file 1 — Additional file 1: Table S1. Study variables. [file 13033_2020_409_MOESM1_ESM.docx]

**TABLE S1**: Study variables.

| **PATIENT VARIABLES** | |
| --- | --- |
| **Sociodemographic variables** | |
| ***Categorical variables*** | |
| **Gender** | Male  Female |
| **Marital status** | Single  Married/Civil partnership/Cohabiting  Separated/Divorced/Widowed |
| **Educational level** | No formal education and/or illiterate  Primary school  Secondary school  Higher education (Bachelor’s degree) |
| Living arrangements | Alone  Original family / other relatives or friends  Own family  Sheltered accommodation  Homeless |
| **Employment status** | Employed  Unemployed  Student  Carer or househusband/housewife  Not working, receiving welfare benefits  Other |
| **Type of area** | Urban  Rural |
| **Within a socioeconomically deprived area** | No  Yes |
| **Primary care centre** | Trinidad  Nueva Málaga  Miraflores  Palma-Palmilla  Ciudad Jardín  Capuchinos  Carlinda  Alameda Perchel  Victoria  Limonar  El Palo  Rincón de la Victoria  Colmenar |
| ***Continuous variables*** | |
| **Age** | |
| **Clinical variables** | |
| **ICD-10 clinical diagnosis** | F20 Schizophrenia  F22 Persistent delusional disorders  F23 Acute and transient psychotic disorders  F25 Schizoaffective disorders  F21, F24, F28, F29 Schizotypal disorder, **Induced delusional disorder,** other non-organic psychotic disorders and unspecified non-organic psychosis |
| **Global level of severity** | Level I (low severity)  Level II  Level III (high severity) |
| **Cardiovascular risk factors (type 2 diabetes mellitus, hypertension, hypercholesterolaemia, obesity and smoking)** | Yes  No |
| **Taking antipsychotic medication** | Yes  No |
| **GENERAL PRACTITIONERS VARIABLES** | |
| ***Categorical variables*** | |
| **Gender** | Male  Female |
| **Specialisation training as a general practitioner (GP)** | Yes  No |
| **Accredited training as a tutor** | Yes  No |
| **Having any residential training student** | Yes  No |
| ***Continuous variables*** | |
| **Age** | |
| **Time to complete the Medical degree** | |
| **Size of patient list** | |
| **Relationship** (level of satisfaction of GPs with their relationship with the community mental health centre (range 7–15; higher scores indicated greater satisfaction with the relationship))^1^. | |
| **Training** (the GPs’ perception of their level of training in mental health, schizophrenia and other psychotic disorders (range 5–15; higher scores indicate greater perceived adequacy of training))^1^. | |
| **Beliefs** (this touched upon erroneous beliefs, stigmas and attitudes regarding mental illness (range 5–12; higher scores indicate more erroneous beliefs and greater stigmatisation))^1^. | |
| ^1^Moreno-Küstner B, Bordallo-Aragon A, Sepúlveda-Muñoz, J. Psychometrics properties of a questionnaire on the attitudes of general practitioners towards mental health (MAPSAM-14). An Psicol-Spain. 2018;34(2):258-263. | |
| **PRIMARY CARE CENTRE VARIABLES** | |
| **Hometown** | 2,500 – 5,000 inhabitants  5,000 – 10,000 inhabitants  10,000 – 15,000 inhabitants  15,000 – 20,000 inhabitants  20,000 – 30,000 inhabitants  More than 30,000 inhabitants |
| **Frequency of mental health care visits in primary care centres** | None  Once a year or less  Between 4 and 6 months  Every 3 months  Every 2 months  Once a month  Twice a month  More than twice a month |
| **Frequency of mental health training sessions in primary care centres** | None  Once a year or less  Between 4 and 6 months  Every 3 months  Every 2 months  Once a month  Twice a month  More than twice a month |
| **Training from mental health services** | Yes  No |
| **Primary care physicians play an active role in managing patients’ mental health** | Completely disagree  Disagree  Neither agree nor disagree  Agree  Completely agree |
| **How would you rate the communications between the primary care centre and community mental health centre?** | Very bad  Bad  Neither good nor bad  Good  Very good |
| **How would you rate the communications of the centre’s primary care physicians and nurses?** | Very bad  Bad  Neither good nor bad  Good  Very good |
| **How would you rate the level of communication between primary care physicians and social workers?** | Not applicable  Very bad  Bad  Neither good nor bad  Good  Very good |
| **Nurses play an active role in managing patients’ mental health** | Completely disagree  Disagree  Neither agree nor disagree  Agree  Completely agree |
| Social workers play an active role in managing patients’ mental health | Not applicable  Completely disagree  Disagree  Neither agree nor disagree  Agree  Completely agree |
