# Supplementary material for: Patterns of primary care among persons with schizophrenia: the role of patients, general practitioners and centre factors
Source: Int J Ment Health Syst. 2020 Nov 10;14:82. doi: 10.1186/s13033-020-00409-z (PMC7653995; doi:10.1186/s13033-020-00409-z)
Supplement: Supplementary file 2 — Additional file 2: Table S2. Categorical variables associated with the number of visits to general practitioners. [file 13033_2020_409_MOESM2_ESM.docx]

**TABLE S2**: Categorical variables associated with the number of visits to general practitioners.

|  | **Mean** | **Median** | **Minimum** | **Maximum** |
| --- | --- | --- | --- | --- |
| **Patient gender**  Women  Men | 17.53  11.25 | 15.5  10.0 | 0  0 | 69  45 |
| **Patient marital status**  Single  Married/Civil partnership/Cohabiting  Separated/Divorced/Widowed | 11.73  19.68  14.90 | 10  15  13 | 0  1  0 | 45  69  34 |
| **Patient educational level**  No formal education and/or illiterate  Primary school  Secondary school  Higher education (Bachelor’s degree) | 18.33  11.57  14.37  11.62 | 15  9  13.5  10 | 0  0  0  0 | 47  46  69  30 |
| **Primary care centre**  Trinidad  Nueva Málaga  Miraflores  Palma-Palmilla  Ciudad Jardín  Capuchinos  Carlinda  Alameda Perchel  Victoria  Limonar  El Palo  Rincón de la Victoria  Colmenar | 17.86  14.57  11.30  14.17  13.20  9  15  9.11  14.70  9.67  17.56  10.82  33 | 18  13  8.5  10  13  8.5  14  7.5  12  6  14.5  9  33 | 0  2  2  0  0  0  11  0  0  0  0  0  33 | 46  27  22  44  47  20  20  35  45  32  69  33  33 |
| **ICD-10 Clinical diagnosis**  F20 Schizophrenia  F22 Persistent delusional disorders  F23 Acute and transient psychotic disorders  F25 Schizoaffective disorders  F21, F24, F28, F29 Schizotypal disorder, **Induced delusional disorder,** other non-organic psychotic disorders and unspecified non-organic psychosis | 13.38  15.81  10  20  8.54 | 11  13  8  19  8 | 0  0  0  1  0 | 69  47  29  46  19 |
| **Cardiovascular risk factors**  Yes  No | 11.06  17.26 | 10  14 | 0  0 | 47  69 |
| **Taking antipsychotic medication**  Yes  No | 10.57  14.85 | 8  13 | 0  0 | 41  69 |
| **Primary care physician active role in managing patients’ mental health**  Neither agree nor disagree  Agree  Completely agree | 11.22  13.67  16.38 | 9  11  14 | 0  0  0 | 33  47  69 |
| **Nurse active role in managing patients’ mental health**  Disagree  Neither agree nor disagree  Agree  Completely agree | 17.56  12.44  13.39  12.44 | 14.5  10  13  10 | 0  0  0  0 | 69  45  47  46 |
| Level of communication between primary care physicians and social workers Neither good nor bad  Good  Very good | 11.28  13.07  15.01 | 9  11  13 | 0  0  0 | 47  46  69 |
